# Intermolecular atom–atom bonds in crystals?^1^

**DOI:** 10.1107/S2052252515002006

**Published:** 2015-02-26

**Authors:** Jack D. Dunitz

**Affiliations:** a Organic Chemistry Laboratory, Swiss Federal Institute of Technology (ETH-Zurich), Zurich, CH-8093, Switzerland

**Keywords:** crystals, intermolecular atom–atom bonds

## Abstract

Some questions are raised concerning the interpretation of distances between atoms of neighbouring molecules in crystals.

The concept of interatomic bonds with more or less characteristic internuclear bond distances and bond energies is firmly established in molecular chemistry. The transfer of this concept to the world of intermolecular arrangements in crystals is appealing and has come into wide usage in discussions of actual and possible crystal structures. However, there are circumstances that render such a transfer questionable and perhaps even untenable, apart from a few special cases, unless we are prepared to use the same word for describing radically different concepts. In crystals, short distances between pairs of atoms in neighbouring molecules – ‘contact’ distances, van der Waals distances – do not necessarily correspond to specific bonding interactions between the atoms concerned. They may indeed sometimes be associated with a lowering of the potential energy associated with a specific attractive force between the atoms concerned – bonding – but they may also be associated with an increase in potential energy and a repulsive force. The close stacking of planar anions, as occurs in salts of croconic acid, may serve as a striking example of the latter. Far from producing a lowering of the crystal energy, this stacking interaction in itself leads to an increase by several thousand kJ mol^−1^ arising from Coulombic repulsion between the doubly negatively charged anions. This large destabilization is, of course, more than compensated in the overall energy balance by the large stabilization arising from Coulombic interactions of the croconate anions with the surrounding cations.

Individual atom–atom pair interactions in crystals may attract attention for one reason or another, but they are seldom structure determining. Rather they emerge from the overall packing of the molecules in the crystal as a whole, involving the interactions of each molecule with all the other molecules in its vicinity.

The interaction energy between any particular pair of molecules in an actual or hypothetical crystal structure can be estimated by quantum mechanical calculations at various levels of complexity or by the *Pixel* method, based on the computed charge density distributions of the individual molecules. Interaction energies obtained by these methods are usually in good overall agreement, allowing for minor systematic discrepancies. Interaction energies for neighbouring molecular pairs in a crystal need not all be negative. Molecules in close proximity in a crystal may attract one another or they may repel one another; only the total energy sum for the crystal as a whole must be negative. There is no reason to assume that the interaction between any particular pair of atoms in neighbouring molecules is bonding simply because the distance between these atoms lies in some particular range. Generally, for any given type of intermolecular atom–atom interaction in a crystal, C–H⋯C, C–H⋯O, C–H⋯F, C–H⋯Cl or C–Cl⋯Cl, *etc*., there is a spread of internuclear distances around the van der Waals radius sum. Atom–atom force fields, which came into use some fifty years ago, are based on atom–atom pair interaction energies as function of internuclear distance. Atom–atom potentials from different force fields differ in many ways – functional form and parametrization – but all agree in their general form. This is a shallow *−ar^−^* ^6^ type potential coupled to a very steep *+br* ^12^ type repulsive term; a gentle downward slope ending in a steep wall. All such potentials agree that atom–atom energies associated with distances much shorter than the sum of the van der Waals radii are positive and associated with repulsion rather than with bonding. The atoms in contact are from the peripheries of neighbouring molecules in the crystal. They hold the molecules apart against the mutual attraction of the more polarizable atoms in the molecular cores.

The main exceptions to these conclusions involve intermolecular hydrogen bonding, a topic that merits special discussion as so much has been written about it over the years. Indeed, hydrogen bonding is the only type of interaction that is important both at the intra- and intermolecular levels. As an example of the former we have the internal hydrogen bond in the hydrogen maleate anion, associated with the unusually large difference of four p*K* units between the two acid strengths of maleic acid (p*K*_a_ = 1.8 and 6.1; compare fumaric acid with p*K*_a_ = 3.9 and 4.4). On the intermolecular side, we have the prime example of the ice crystal, with its remarkably high melting point (compared with dihydrogen sulfide), held together entirely by O–H⋯O hydrogen bonds, as well as countless other examples.

Whereas the energies associated with the weakest covalent bonds in molecules are of the order of 150 kJ mol^−1^, energies associated with intermolecular pair interactions seem to have no lower limit. Should one then attempt to set a limit? It would not be easy. In any case, there is a fundamental difference between the two types of bonding, intra- and intermolecular. The energy of a particular intramolecular bond can sometimes be measured directly, at least in principle. By experiment or by calculation, the molecule in question can be dissociated into two discrete fragments by severing the bond, and the energy change in this process can be measured or calculated by some suitable procedure. The result can be identified with the bond breaking energy. Linus Pauling’s *Nature of the Chemical Bond* describes many examples. For intermolecular bonding, the two molecules in question can in principle be drawn apart along the direction of the putative intermolecular bond, but the energy change associated with this process can no longer be identified exclusively with the separation of the two atoms concerned. Other intermolecular pair distances must change in the separation process, so the energy change cannot be identified with any particular pair.

As an extension of Richard Bader’s *Atoms in Molecules* approach, evidence for specific bonding interactions between pairs of atoms in neighboring molecules, such as those in molecular crystals, has been adduced. The evidence in question comes from the extension of bond path analysis from the intramolecular to the intermolecular level. A bond path (in the Bader sense) is a line along which the electron density *ρ*(***r***) connecting two atomic nuclei is a maximum with respect to any neighboring line; the electron density and its curvature at the saddle point along the bond path provide a measure of the strength of the electronic ‘glue’ holding the atoms together along the bond path. Covalent bonds in molecules yield characteristic bond paths. For the intermolecular case, the matter is more controversial. Since the electron density between molecules in crystals is orders of magnitude less than the electron density between bonded atoms in molecules, intermolecular bond paths are far weaker and more difficult to distinguish from the background. Nevertheless, countless publications claiming the observation of such intermolecular bond paths in electron density distributions derived from experimental X-ray diffraction measurements of molecular crystals have appeared in the scientific literature, and the question arises as to whether such results provide compelling evidence of intermolecular atom–atom pair bonding or whether they could just as well arise from simple overlapping of atomic electron densities. For example, it has been shown by Mark Spackman and Carlo Gatti that bond paths for hydrogen bonds derived from experimental electron densities in crystals are close to those obtained by simple addition of non-interacting, overlapping, spherical atom electron densities. If bond paths for hydrogen bonds are in question, what confidence can we have in claims for the observation of bond paths for other types of intermolecular contact?

The purpose of this little essay is to pose the question: should the observation of short distances between pairs of atoms on the peripheries of different molecules in crystals be regarded as evidence of specific intermolecular bonding between the atoms concerned? And if the answer is not yes but no or perhaps or sometimes: how are we to distinguish the bonding atom–atom interaction from the energetically neutral or *anti*-bonding type? Do we need another IUPAC commission to decide?

